# Hypothesis: Mutations and Immunosurveillance in Obesity-Associated Colorectal Cancer

**DOI:** 10.7150/jca.76052

**Published:** 2022-08-08

**Authors:** Darina Lazarova, Michael Bordonaro

**Affiliations:** Department of Medical Education, Geisinger Commonwealth School of Medicine, 525 Pine Street, Scranton, PA 18509, USA.

**Keywords:** Colon cancer, obesity, mutations, BMI, cell signaling

## Abstract

Tumorigenesis typically requires the accumulation of several driver gene mutations; therefore, there is a mutation threshold for the completion of the neoplastic process. Obesity increases the risk of cancer, and we have proposed that one mechanism whereby obesity raises the risk of microsatellite stable (MSS) colon cancer is by decreasing the mutation threshold. Therefore, obese MSS colon cancer patients should exhibit fewer driver gene mutations compared to normal body-mass index (BMI) patients. Our hypothesis is supported by results from analyses of The Cancer Genome Atlas (TCGA) data, which revealed that cancer genomes of obese MSS colon patients exhibit both fewer somatic mutations and fewer driver gene mutations. These findings could be explained by the high levels of obesity-associated cytokines and factors, the signaling pathways of which substitute for the additional driver gene mutations detected in normal-weight MSS colon cancer patients. Therefore, obesity-induced aberrant cell signaling might cooperate with initiating driver gene mutations to promote neoplastic development. Consistent with this possibility, we observed a lower number of *KRAS* mutations in high-BMI MSS colon cancer patients. This paper extends our hypothesis to address the interactions between obesity, immune surveillance in neoplastic development, and colorectal cancer (CRC) risk. A better understanding of these interactions will inform future preventive and therapeutic approaches against MSS CRC. We propose that the individual variations in the major histocompatibility class 1 (MHC-1) genotype interact with obesity to shape the tumor mutational landscape. Thus, the efficiency of the immune surveillance mechanisms to select against specific mutations may depend on both the MHC-1 genotype variant and the BMI of an individual. A high BMI is expected to reduce the number of driver gene mutations required to evade the MHC-1 surveillance mechanism and support an accelerated cancer progression.

## Introduction

Obesity, a worldwide epidemic that is particularly prevalent in developed countries such as the U.S. (i.e., over 42 percent of U.S. Americans are obese), is associated with an increased risk of colorectal cancer (CRC) 1-6. Obesity among children and adolescents 7 is likely a major contributor to the recent sustained increase in CRC incidence observed in young adults aged 20-34 8. Considering the typical 10 - to 20 - year period from the initiation of colonic neoplasia to the establishment of a carcinoma, CRCs diagnosed in young adults are likely initiated in elementary school- and middle school-age children [9 and refs therein].

Several mechanisms have been proposed to explain the observation that obesity is linked to a reduced number of driver gene mutations in certain cancers 9-11. The role of MHC-1 genotypes in influencing the oncogenic mutational landscape 12 may also affect the obesity-shaped mutation burden in cancer. For example, an HLA mutation has been linked to an increased mutational burden 12.

MHC-1 genotypic variation is driven by several factors. The three main MHC-1 genes, *HLA-A, HLA-B*, and *HLA-C* are highly polymorphic, with thousands of possible alleles 12. There are also non-classical genes, such as *HLA-G*, *HLA-E*, and *HLA-F* that are overexpressed in many cancers 13. These variants exhibit functional differences; for example, A*25:01, B*15:01, and C*15:2 have broad peptide binding profiles 14 and A*02:01 has been predicted to bind a variety of mutant proteins and this has been supported by experimental data utilizing mass spectrometry-derived peptides 12. Furthermore, the non-classical HLAs overexpressed in cancer have been shown to bind inhibitory receptors on effector cells and repress the function and enhance the apoptosis of these cells, contributing to immunosuppression 13.

Differential HLA expression has been observed in obesity 15. In addition, obesity-related chronic inflammation promotes the formation of Myeloid Derived-Suppressor Cells that repress immune function, including through a mechanism of inhibiting CD8+ T cells 16. The inhibition of T cell function can synergize with functional differences of different HLA types, and their levels of expression, in the process of tumorigenesis. Thus, MHC-1 genotypic variations 12 can interact with obesity-related factors to influence the spectrum of driver gene mutations in cancer.

## Interpretations of the original hypothesis relevant to the current paper

According to the multiple-hit hypothesis for CRC and other types of cancer, tumorigenesis requires the accumulation of several mutations. Therefore, there is a mutation threshold for the establishment of cancer. We have previously proposed that one mechanism whereby obesity raises the risk of microsatellite stable (MSS) colon cancer is by decreasing the mutation threshold 9. If this hypothesis is true, then compared to normal-weight individuals, obese individuals develop MSS colon cancer with fewer driver gene mutations. The hypothesis is supported by analyses of The Cancer Genome Atlas (TCGA) data. The analyses revealed that the cancer genomes of obese MSS colon cancer patients exhibit fewer somatic mutations and fewer driver gene mutations compared to normal-weight MSS colon cancer patients 9. This statistically significant difference is likely due to the presence of obesity-associated factors (e.g., cytokines, hormones, etc.) and their proliferative and cell survival signaling that substitute for the signaling supported by driver gene mutations in normal-weight patients. Therefore, obesity-induced aberrant cell signaling cooperates with initiating driver gene mutations to promote carcinogenesis. Consistent with this hypothesis, we observed a lower number of *KRAS* mutations detected in high-BMI colon cancer patients 9. In addition, the tumor suppressor gene *PTEN* was mutated less frequently in high-BMI colon cancer patients 9. The original analysis had several limitations, among which were the small cohort size, the lack of data on possible confounding factors (e.g., BMI at cancer diagnosis may not be representative of the long-term BMI history of the patient), and the exclusion of non-coding sequences from the analyses, as the data were based on whole-exome sequencing. Assuming that the results are confirmed by future extended TCGA analyses, what conclusions can be currently made?

Plausible mechanisms 9-11 that could reduce the number of driver gene mutations in obesity include (a) obesity-induced survival and proliferative signaling pathways (e.g., ERK and AKT) that substitute for driver gene mutations activating the same pathways in normal-weight patients, (b) obesity-induced changes in the gut microbiome 10 and its downstream pathways that substitute for driver gene mutations, (c) obesity-induced epigenetic changes that substitute for driver gene mutations, and (d) stem cell numbers that are increased in obesity [11 and refs. therein]. Most likely, a combination of these mechanisms plays a role in the association between obesity and the reduced number of driver gene mutations observed in high-BMI MSS colon cancer patients. There might be also confounding age-related factors that impact the association between obesity and CRC risk. For example, an increased number of febrile episodes in a person's lifetime and a higher number of years on medications for obesity-related diseases could lower cancer risk 11. Tumor immune surveillance may also play a crucial role in obesity-associated MSS CRC, shaping the cancer mutational landscape in a manner additive or synergistic to the effects of obesity.

## Extending the original hypothesis to include tumor immune surveillance in obesity

There is convincing evidence that individual variations in MHC-1 genotypes influence the combinations of driver gene mutations in cancer 12. The MHC molecules present proteins from inside the cells to the cells' surface to facilitate the detection of mutated and foreign proteins by the T cells. Poor presentation of mutated driver gene products by the MHC-1 molecules does not allow the T cells to recognize and detect the mutated peptides; therefore, such mutations are tolerated (i.e., they are not blocked) by the immune system and are represented at a high frequency in tumors. As a result, different levels of immune surveillance efficacy, determined by peptide antigenicity and MHC-1 genotype variations, influence the tumor mutation profile in neoplastic development.

The immune surveillance efficacy based on MHC-1 molecules also differs during the stages of neoplastic progression. It is thought that the MHC-1 surveillance is prevalent during the early stages of tumorigenesis when neoplasms have not yet acquired effective immunosuppressive mechanisms 12. At later stages of neoplastic development, under immunosuppressive conditions, the tumors expand rapidly and may acquire a wider range of mutations, and some of these might be antigenic driver gene mutations, however, most of these might be passenger mutations that do not provide a selective advantage to the mutant cells 12. According to this model, driver gene mutations that are more frequently observed in tumors are less antigenic, and such mutations produce peptide sequences that are poorly presented by the MHC 12. Conversely, driver gene mutations that are more antigenic would be preserved in the immunosuppressive environment of late neoplastic development, and/or would be found in patients whose MHC-1 genotypes are ineffective in presenting the products of the mutated genes 12. Therefore, whereas antigenic mutations would be less frequently observed among the general population of cancer patients, they would be observed more frequently in a subset of patients with less efficient immune function. In summary, the tumor profile of each cancer patient is affected by their unique MHC-1 genotype 12.

Obesity and MHC-1 genotype variations may interact in different ways. High BMI reduces the number of driver gene mutations necessary for neoplastic development 9. However, obesity also impairs immune function and reduces the effectiveness of tumor immune surveillance [17, 18, and refs therein], and tumor-associated adipocytes can alter the tumor microenvironment to modulate immune function 19. Therefore, in obesity, fewer driver gene mutations are required for neoplastic development and fewer driver gene mutations might be blocked from being represented in cancer due to the obesity-suppressed MHC-1 immune surveillance. If obesity uniformly impairs the surveillance function of all MHC-1 genotype variants, then the accumulation of the few required driver gene mutations would be accelerated to the same extent in all obese individuals. However, if the MHC-1 genotype variants exhibit different levels of sensitivity to the immunosuppressive effects of obesity, then MHC-1 genotype variants with low sensitivity to obesity-induced immunosuppression would be relatively effective in blocking driver gene mutations in early neoplastic development. In contrast, MHC-1 genotype variants with high sensitivity to obesity-induced immunosuppression would be less effective in constraining driver gene mutations, and therefore, individuals with such MHC-1 variants are predicted to develop CRC in an accelerated fashion. Furthermore, in obesity, the impaired MHC-1 immune surveillance is combined with the necessity of fewer driver gene mutations for neoplastic progression. Therefore, the interaction between obesity and MHC-1 genotype variants may result in an additive or synergistic acceleration of the neoplastic process.

In our previous analyses of TCGA data on MSS colon cancer mutations 9, overweight and obese patients were diagnosed at a younger age than normal-weight patients; however, the differences were not statistically significant (data not shown). The existence of MHC-1 genotype variations may in part explain these results. For example, high-efficiency MHC-1 genotype variants, a high number of febrile episodes, fewer years of high BMI, and more years on medications for manifestations of metabolic syndrome are all factors that might extend the time required for neoplastic progression even in the context of obesity.

## Testing the hypothesis

TCGA data analyses have revealed that the high BMI of MSS colon cancer patients correlates with a lower number of driver gene mutations 9. To test the hypothesis proposed here, future analyses of TCGA data should be expanded to include MHC-1 genotype status. We expect statistically significant differences in the numbers of CRC mutations, particularly driver gene mutations when considering MHC-1 genotypes as a variable in conjunction with BMI. Statistical tests, such as the determination of synergy factor 20, would evaluate possible synergistic interactions between BMI, MHC-1 status, and patient mutation profile. The analyses should also ascertain how the combination of the three factors BMI, MHC-1 genotype variants, and CRC mutation profile impacts patient characteristics such as age of cancer diagnosis and overall survival. We expect that the ability to predict the tumor mutation spectrum based on the MHC-1 genotype variants 12 would be affected in a statistically significant manner by the patient's BMI. Thus, the MHC-1 genotype constrains the possible mutation profiles of the patients, whereas a higher BMI reduces the total number of driver gene mutations represented in a tumor by decreasing the necessity for specific types of mutations.

The interactions between BMI and MHC-1 genotype variants should be tested for synergy because the surveillance based on MHC-1 genotype variants limits the mutational spectrum by blocking antigenic mutations; however, high BMI suppresses this immune surveillance, and, therefore, accelerates the accumulation of the few driver gene mutations necessary for neoplastic progression in obesity. We expect the mutation profile of patients with MSS CRC to be determined by (a) what alterations in cell signaling and gene expression are required for tumorigenesis, (b) which mutations are tolerated by the immune surveillance mechanism, (c) which mutations are substituted for by the effects of obesity-induced cytokines and factors, and (d) the degree to which immune function is suppressed by high BMI. We expect statistically significant effects of MHC-1 genotype variant status and BMI variations on the mutation profiles. The type, degree, and direction of these effects would need to be determined empirically.

In addition to the data analyses of cancer patient cohorts, studies on weight gain and immune function (or the lack thereof) in experimental mouse models of CRC can be employed to ascertain changes in the mutation profile of tumors based on the interplay of animal obesity and immune status. The correlations observed in animal models are expected to confirm the observations from human cohorts. Animal models would allow for controlled studies, including investigation of molecular and cellular mechanisms, to better understand the underlying associations, and to test potential preventive and therapeutic approaches that target obesity-related cancer in a manner dependent upon tumor surveillance. Differences between the mutation profiles of CRC patients and animal models of cancer, however, may limit the degree to which animal studies can fully explain the human condition. Despite these limitations, animal models of CRC, particularly when used to dissect molecular mechanisms, can serve as a useful addition to the human cohort data.

## Implications of hypothesis

The design of treatment options for high-BMI MSS CRC patients must consider the mutation profile of individual cancers and target obesity-induced cell survival and proliferative signaling. Otherwise, obesity-deregulated cell signaling would render current standard therapeutic regimens relatively ineffective. For example, in an individual tumor, AKT1/2 signaling might not be activated by a mutation; however, it might be activated by obesity. Therefore, a pharmacological intervention that includes an inhibitor of the pathway would be beneficial for high-BMI cancer patients.

Another therapeutic consideration is based on the knowledge that obesity establishes an immunosuppressive environment during neoplastic development. Therefore, compared to normal-weight individuals, obese individuals are likely to acquire a higher proportion of driver gene mutations that are antigenic. Presumably, in normal-weight patients, the antigenic mutations are blocked at least in the early stages of neoplastic development, when MHC-1 surveillance is functional and effective. In obesity, due to the increased immunosuppression, such antigenic mutations may not be blocked even in the early stages of neoplastic development. The increased availability of antigenic cancer mutations may render obese MSS CRC patients suitable for immune therapy; however, this treatment approach must be combined with weight reduction to decrease the obesity-imposed immunosuppression. This reasoning is supported by the findings that reduced cancer incidence in obese individuals and favorable cancer outcomes in cancer survivors are associated with a negative energy balance 21. Until recently, MSS CRC has been considered resistant to immunotherapy approaches; however, combination pharmacological approaches might change the current standard of care 22.

**Figure 1:**
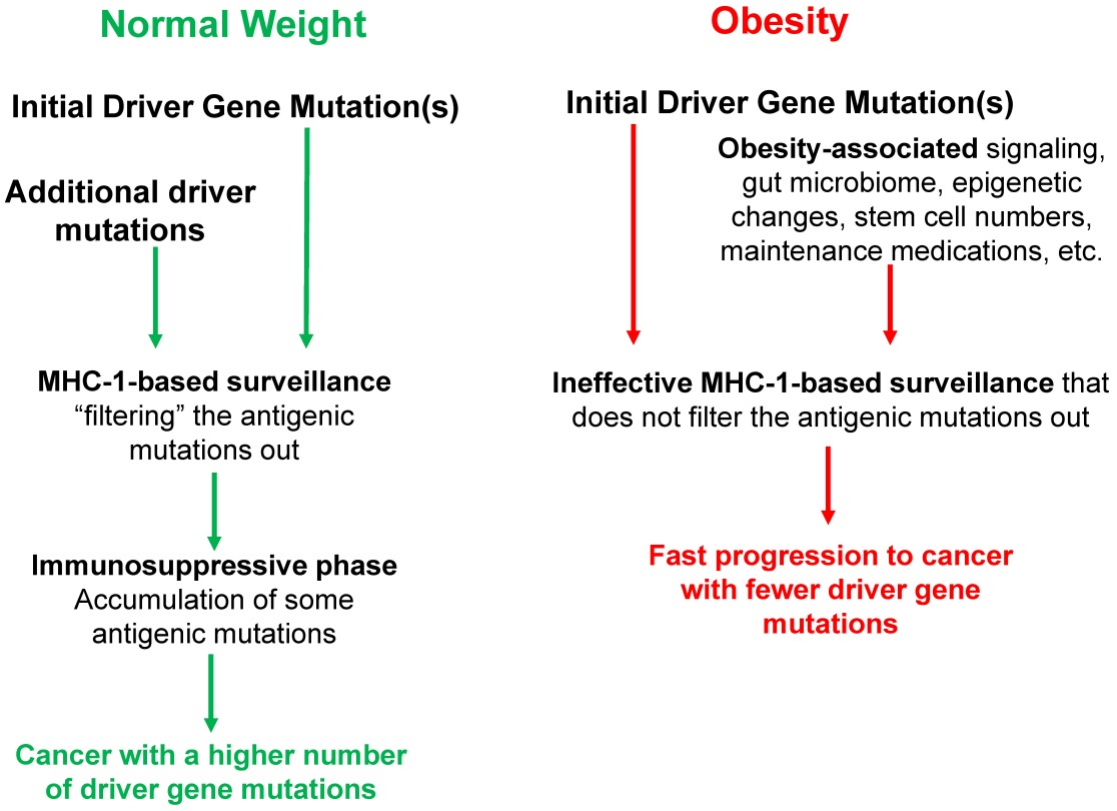
** The interplay between BMI and MHC-1-based surveillance impacts the mutation landscape of cancer**. Initial driver gene mutations start the neoplastic process. In obese individuals, the activation of cell survival and proliferative signaling pathways, altered gut microbiome, epigenetic changes, increased stem cell numbers, maintenance medications, and febrile episodes (among others) influence the process of tumorigenesis, affecting the number and type of driver gene mutations required for progression to CRC and the age of cancer diagnosis. MHC-1 genotypes create immune surveillance filters with different efficiency to shape the mutation landscape of cancer. The immune surveillance is suppressed by obesity, thus further differentiating the mutation profiles of normal-BMI and high-BMI individuals. During the immunosuppressive late stages of neoplastic development, some antigenic mutations are no longer selected against and can accumulate particularly in normal-weight individuals, and such individuals usually require a higher number of driver gene mutations for neoplastic progression. All of these factors, as well as lifestyle differences (not included here), affect the cancer mutation landscape, and the age of cancer diagnosis in normal-BMI *versus* high-BMI individuals.

Concerning prevention, weight control is required, and individuals with consistently higher than normal BMI should start CRC diagnostic screening at younger ages than their normal-weight counterparts. MHC-1 genotype variants should be included in the calculations to determine the relative degrees of CRC risk, combined with BMI and other lifestyle and life history factors (Fig. 1).

## Conclusion

We have previously reported that obesity is associated with fewer driver gene mutations in colon cancer 9. Subsequently, we proposed mechanisms for this observation and the age-dependent changes in cancer risk 11; these are discussed above (i.e., section “Interpretations of the original hypothesis relevant to the current paper”). These previous reports concluded that obesity reduces the number of driver gene mutations in colon cancer through a variety of mechanisms that interact with factors associated with the age of the patients. In contrast, the current hypothesis primarily focuses on the role of the immune system and its interplay with the aforementioned mechanisms, in determining the number and type of driver gene mutations. We propose that MHC-1 genotype variations and patient BMI influence the efficiency of the immune surveillance mechanisms, resulting in an association between high BMI and a reduced number of driver gene mutations required to evade the MHC-1-dependent immunosurveillance mechanism. Therefore, the current manuscript extends the original proposal to include the innate patient characteristics (e.g., the MHC-1 genotype variants) along with previously discussed lifestyle and external factors (e.g., obesity, febrile episodes, maintenance medication, diet-influenced changes in the gut microbiota).

**Figure 2:**
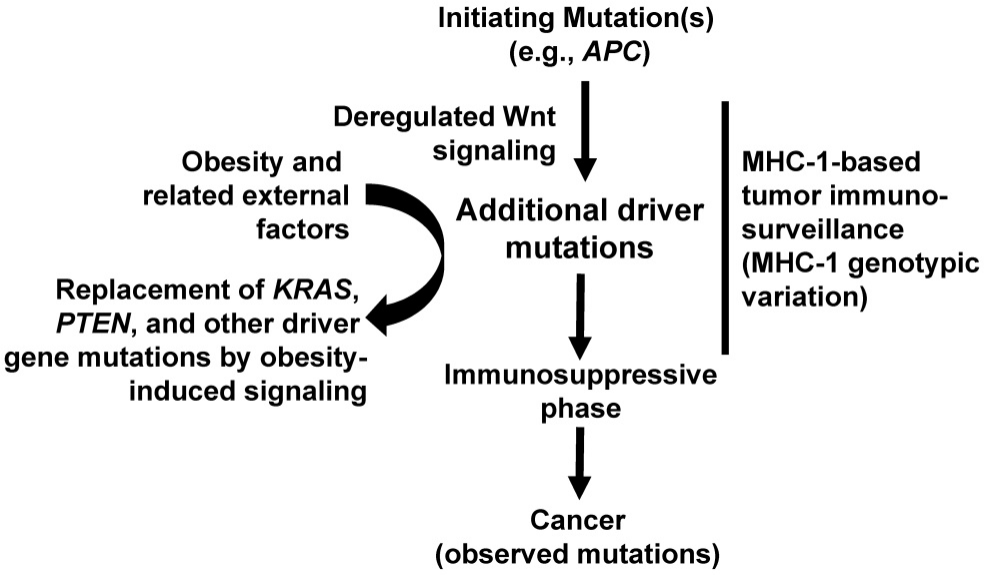
** Timeline of neoplastic events in MSS colorectal cancer and our hypothesis.** An initiating mutation (typically in *APC*) deregulates Wnt activity. Later mutations may reinforce this deregulation and may be more frequent in obesity. However, subsequent driver gene mutations (e.g., *KRAS*, *PTEN*) are less frequent in obesity because the downstream effects of these mutations are substituted by obesity-induced cell growth and survival pathways. Several external factors (e.g., lifestyle, obesity, and other factors synergizing with obesity) influence the number and type of driver genes in individual tumors. After the first initiating event(s), tumor immunosurveillance, influenced by the MHC-1 genotypic variation, acts as a “sieve” to affect the spectrum of mutations in surviving tumor cells.

With respect to molecular and cellular mechanisms whereby the hypothesis is associated with the development of colorectal cancer, we refer back to the spectrum of driver gene mutations exhibited in obesity 9. For example, in MSS colon cancer, a higher BMI is associated with a decreased mutation frequency of *KRAS* and *PTEN*. This is likely due to the obesity-induced cell survival and proliferation pathways (e.g., such as ERK and AKT) that bypass the requirement for mutations in these pathways. On the other hand, consistent with its typical role in initiating most colorectal cancer, in MSS cancer, the mutation frequency of *APC* is not decreased in high-BMI patients. Indeed, the data suggest that high BMI is associated with colorectal cancer with increased Wnt signaling. Therefore, we can envision a scenario in which a mutation of *APC* (and/or other Wnt signaling-related genes) results in deregulated Wnt activity and the initiation of colorectal tumorigenesis. After cancer initiation through Wnt signaling deregulation, *KRAS* and *PTEN* mutations in normal-weight individuals can be advantageous; however, such mutations in high-BMI individuals are less advantageous because the survival and proliferative pathways supported by such mutations are substituted by obesity-induced signaling. Importantly, the spectrum of post-*APC* driver gene mutations is influenced not only by which driver gene mutations are made redundant by obesity-induced cell signaling, but also by which neoplastic cells survive the tumor immunosurveillance, which is affected by the MHC-1 genotypic variation. These interpretations are summarized in Fig. 2.

The perspectives discussed here are likely to apply not only to CRC but also to other cancer types that exhibit an obesity-associated increase in incidence among younger adults 23. Obesity-related conditions, such as type 2 diabetes, which are linked to changes in cell signaling and tissue metabolism, are also becoming more prevalent among young adults, concomitant with overweight and obesity in these age cohorts 19. Such morbidities likely contribute, in a causal mechanistic manner, to the increased cancer burden among younger individuals 23, possibly by lowering the number of driver gene mutations required for tumorigenesis and imposing an immunosuppressive tumor environment. Whereas in normal-weight individuals, an immunosuppressive environment is developed in later stages of neoplastic development, in obesity, the evasion of immune surveillance is likely to take place in both the early and late stages of neoplastic development 24. Therefore, in obesity, the interplay between obesity-induced signaling, accumulating mutations, and suppressed immune surveillance likely affects the entire progression from benign to malignant neoplasm. Understanding the interplay between obesity and MHC-1 genotype variants will assist in the more accurate prediction of cancer risk for all individuals, and in their stratification for different targeted prevention programs that must include dietary intake adjustments, weight control, and physical activity.

In conclusion, an understanding of the mechanisms involved in the associations illustrated in Figures 1 and 2 will optimize the design of preventive and therapeutic strategies against cancer for individuals with high BMI. For example, it has been demonstrated that mice with diet-induced obesity exhibit increased tumorigenic Wnt signaling levels and that this effect is decreased by genetic suppression of the immune system through the ablation of tumor necrosis factor-alpha 25. In this example, the knowledge of these complex interactions would influence the preventive and therapeutic approaches that target Wnt signaling. Immune surveillance may also be indirectly influenced by infections accompanied by febrile episodes, the occurrence of which affects cancer risk 11. This consideration may influence decisions on how to approach the treatment of febrile episodes, particularly in individuals with a high risk of cancer.

Future research should ascertain how obesity substitutes for, and/or augments, driver gene mutations through altered cell signaling. Such studies should also examine how obesity-related cancer signaling is influenced by the expression of gene variants that modulate the immune system, the expression of MHC-1, and the tumor immune evasion 26, 27. Emerging data support the concept that obesity-related cancer derives from complex interactions between deregulated cell signaling and immune response to tumors, the latter of which is modulated in part by the expression of MHC-1 gene variants. Controversy still exists concerning the association between the MHC-I genotype and the cancer mutational landscape 28, and the experimental approaches proposed here can assist in ascertaining the role of MHC-1 variability. The acquired knowledge can assist in developing approaches that restore MHC-1 expression and function to promote better tumor immunosurveillance 29.

## Abbreviations

CRC: colorectal cancer
TCGA: The Cancer Genome Atlas
MSS: microsatellite stable
MSI: microsatellite instability
BMI: body mass index
MHC: major histocompatibility complex

## References

[2] Wolin KY, Carson K, Colditz GA (2010). Obesity and cancer. Oncologist.

[3] Limburg PJ, Anderson KE, Johnson TW (2005). et al. Diabetes mellitus and subsite-specific colorectal cancer risks in the Iowa Women's Health Study. Cancer Epidemiol Biomarkers Prev.

[4] Takahashi H, Yoneda K, Tomimoto A (2007). Lifestyle-related diseases of the digestive system: colorectal cancer as a lifestyle-related disease: from carcinogenesis to medical treatment. J Pharmacol Sci.

[5] Aleksandrova K, Nimptsch K, Pischon T (2013). Obesity and colorectal cancer. Front Biosci.

[6] Bardou M, Barkun AN, Martel M (2013). Obesity and colorectal cancer. Gut.

[7] Ogden CL, Carroll MD, Kit BK (2014). Prevalence of childhood and adult obesity in the United States, 2011-2012. JAMA.

[8] Bailey CE, Hu CY, You YN Increasing Disparities in the Age-Related Incidences of Colon and Rectal Cancers in the United States, 1975-2010 JAMA Surg. 2015; 150: 17-22.

[9] Bordonaro M, Lazarova D (2015). Hypothesis: Obesity is Associated with a Lower Mutation Threshold in Colon Cancer. J Cancer.

[10] Ley RE (2010). Obesity and the human microbiome. Curr Opin Gastroenterol.

[11] Lazarova DL, Bordonaro, M (2021). Multifactorial causation of early-onset colorectal cancer. J. Cancer.

[12] Marty R, Kaabinejadian S, Rossell D (2017). MHC-I genotype restricts the oncogenic mutational landscape. Cell.

[13] Kochan G, Escors D, Breckpot K, Guerrero-Setas D (2013). Role of non-classical MHC class I molecules in cancer immunosuppression. Oncoimmunology.

[14] Zaituoua AJ, Kaur A, Raghavan M (2020). Variations in MHC class I antigen presentation and immunopeptidome selection pathways. F1000Res.

[15] Fabsitz RR, Nam JM, Gart J (1989). HLA associations with obesity. Hum Hered.

[16] Ostrand-Rosenberg S (2018). Myeloid derived-suppressor cells: their role in cancer and obesity. Curr Opin Immunol.

[17] Yang H, Youm YH, Vandanmagsar B (2009). Obesity accelerates thymic aging. Blood.

[18] Cramer DW, Finn OJ (2011). Epidemiologic perspective on immune-surveillance in cancer. Curr Opin Immunol.

[19] Tabuso M, Homer-Vanniasinkam S, Adya R (2017). Role of tissue microenvironment resident adipocytes in colon cancer. World J Gastroenterol.

[20] Cortina-Borja M, Smith AD, Combarros O (2009). The synergy factor: a statistic to measure interactions in complex diseases. BMC Research Notes.

[21] Clifton KK, Ma CX, Fontana L, Peterson LL (2021). Intermittent fasting in the prevention and treatment of cancer. CA Cancer J Clin.

[22] Wang C, Fakih M (2021). Targeting MSS colorectal cancer with immunotherapy: are we turning the corner?. Expert Opin Biol Ther.

[23] Sung H, Siegel RL, Rosenberg PS (2019). Emerging cancer trends among young adults in the USA: analysis of a population-based cancer registry. Lancet Public Health.

[24] Mascaux C, Angelova M, Vasaturo A (2019). Immune evasion before tumour invasion in early lung squamous carcinogenesis. Nature.

[25] Guo C, Kim SJ, Frederick AM (2019). Genetic ablation of tumor necrosis factor-alpha attenuates the promoted colonic Wnt signaling in high fat-induced obese mice. J Nutr Biochem.

[26] Kearney CJ, Vervoort SJ, Hogg SJ (2018). Tumor immune evasion arises through loss of TNF sensitivity. Sci Immunol.

[27] Garancher A, Suzuki H, Haricharan S (2019). IMMU-03. Tumor necrosis factor overcomes immune evasion in p53-mutant medulloblastoma, Neuro-Oncology.

[28] Kherreh N, Cleary S (2022). Seoighe, C. No evidence that HLA genotype influences the driver mutations that occur in cancer patients. Cancer Immunol Immunother.

[29] Garrido F, Apgsiauri N, Doordujin EM (2016). The urgent need to recover MHC class I in cancers for effective immunotherapy. Curr Opin Immunol.

